# Reproductive Outcome following Hysteroscopic Monopolar Metroplasty: An Analysis of 203 Cases

**Published:** 2013-09-18

**Authors:** Ensieh Shahrokh Tehraninejad, Firouzeh Ghaffari, Nadia Jahangiri, Mansoureh Oroomiechiha, Mohammad Reza Akhoond, Elham Aziminekoo

**Affiliations:** 1 Department of Endocrinology and Female Infertility at Reproductive Biomedicine Research Center, Royan Institute for Reproductive Biomedicine, ACECR, Tehran, Iran; 2 Department of Obstetrics and Gynecology, Tehran University of Medical Sciences, Tehran, Iran; 3 Vali-e-Asr Reproductive Health Research Center, Tehran University of Medical Sciences, Tehran, Iran; 4 Department of Epidemiology and Reproductive Health at Reproductive Epidemiology Research Center, Royan Institute for Reproductive Biomedicine, ACECR, Tehran, Iran

**Keywords:** Uterine Septum, Hysteroscopic Metroplasty, Infertility, Reproductive Outcome, Assisted Reproductive Techniques

## Abstract

**Background:**

The aim of this study was to evaluate the reproductive outcome of women with history of infertility or recurrent miscarriage following hysteroscopic septum resection.

**Materials and Methods:**

This was a retrospective descriptive study performed on 263 patients, among whom 248 patients were infertile (79% with primary infertility and 21% with secondary infertility) and 15 patients presented with histories of recurrent miscarriage (three or more miscarriages) between 2005 and 2009. All participants underwent hysteroscopic septum resection using monopolar knife electrode. The main outcome measure was reproductive outcome after hysteroscopic metroplasty.

**Results:**

The septum was completely removed during the first hysteroscopy in 242 (92%) patients. A residual septum was seen in 21 (8%) patients who required a second sitting of surgery. Three cases were complicated by minor perforations which required no further interventions. One operation complicated with bleeding which was controlled by a Foley catheter. There were no cases of postoperative Asherman’s syndrome. Postoperatively, out of 263 patients, outcomes of 203 individuals were analyzed. According to the results, the miscarriage rate reduced significantly from 20.2 to 4.9%. Postoperative ectopic pregnancy rate and preterm labor were lower than prior to septum resection. Term deliveries increased significantly from 2.5 to 33.5%.

**Conclusion:**

Hysteroscopic septum resection is a safe and effective method for patients with history of infertility or recurrent miscarriage.

## Introduction

The first hysteroscopic septum resection was performed in 1974, and the first successful outcome following this procedure was in 1981 (1, 2). Uterine septum is the most common congenital uterine malformation with an incidence of 2-3% (3-5), often causing spontaneous miscarriage (6, 7) or premature delivery in 5 to 25% of cases (8, 9). A septate uterus is also seen in about one-third of women with recurrent miscarriages who are diagnosed with Mullerian anomaly (10). The reproductive history of recurrent miscarriage or fetal loss is considered an important indication for uterine septum treatment (11-13). Alternatively, the effect of a sub-septate uterus on women’s fertility in the absence of a bad obstetric history is still a debatable subject (13). The side effects of abdominal surgery, which include the risk of both pelvic adhesions and cesarean delivery, are reduced with hysteroscopy, thereby it has been recognized as a favorable and safer therapeutic procedure for removal of a uterine septum (1, 11, 14-16). The present study aims to evaluate reproductive outcome following septum resection by hysteroscopy in women with history of infertility or recurrent miscarriage.

## Materials and Methods

### Patient selection and protocol

This retrospective study included 263 patients who underwent hysteroscopic septum resection from April 2005 to 2009. Since Royan Institute is considered as a referral center for infertility, 248 out of 263 patients were infertile (79% with primary infertility and 21% with secondary infertility) and 15 patients presented with histories of recurrent miscarriage (three or more miscarriages). The mean age of participants was 30.1 ± 5.9 years. The study was approved by the Institutional Review Board at Royan Institute Research Center and the Royan Ethics Committee. Written informed consent had been obtained from all patients in order to use the data for future scientific research. Septum diagnosis was made during the routine infertility work-up by hysterosalpingography and hysteroscopy. Furthermore, laparoscopy was performed for differential diagnosis of septate and bicornuate uterus. Guidelines from the Society for Reproductive Medicine (17) were applied for classification of the septa into the following two classes: i. Va (complete septate uterus) and ii. Vb (partial septate uterus).

The sole causative factor for infertility is septate uterus which was found in 58 (23.4%) out of 248 patients, while the remaining 190 (76.6%) patients presented with other causes for infertility. A review of the reproductive histories of these 263 patients prior to septum resection showed the following conditions for 155 pregnancies: 127 cases (81.9%) with spontaneous miscarriages, 8 cases (5.2%) with ectopic pregnancies, 11 cases (7.1%) with preterm labor, and 9 cases (5.8%) with full term deliveries. There were 196 patients who had previously undergone hysterosalpingography, in which a uterine septum was detected in 157 (80.1%) of them. All three surgeons, involving in the study, were skilled in performing hysteroscopic septum resections. Three months after the hysteroscopic metroplasty, the patients with an indication for *in vitro* fertilization (IVF), intracytoplasmic sperm injection (ICSI) or intrauterine insemination (IUI) according to departmental protocol underwent controlled ovarian hyperstimulation. After the surgery, patients with >12 months of unprotected intercourse were followed up by phone. These patients were asked about their desire to become pregnant, pregnancy status and outcomes.

### Surgical procedure

The hysteroscopic septum resection was performed in the early proliferative phase under general anesthesia. No medical therapy was prescribed prior to procedure such as oral contraceptive pills (OCP), danazol or gonadotropin agonist. For complete septum, simultaneous laparoscopy was performed to rule out a bicornuate uterus.

The cervix was dilated to 10 mm after which a 7mm rigid hysteroscope (model 26050 EG; Karl Storz, Tuttlingen, Germany) was introduced into the cervix. The uterine cavity was distended with 1.5% glycin (Shahid Ghazi Pharmaceutical Co. Tabriz, Iran). The resection began from the lower margin of the septum with a monopolar knife electrode (Karl Storz, Germany) and continued with a progressive horizontal incision in the midline. The incision was considered as a complete procedure when the ostia were clearly visible under panoramic view. There were 18 cases with vaginal septum. In these cases, the vaginal septum was removed using Mets scissors (Aesculap Inc, Germany) followed by suturing after which the hysteroscopic septum resection was performed. 

All patients postoperatively received oral conjugated estrogens (Aboureihan, Iran) at a dosage of 1.25 mg per day for two months in addition to 10 mg medroxyprogesterone acetate (5mg bid; Aboureihan, Iran) per day for the last 10 days per month of estrogen therapy. In order to evaluate the procedure effectiveness, a follow-up hysterosalpingogram (HSG) was performed after two months. If the result of HSG confirmed an inadequate resection, the residual tissue was removed during the next hysteroscopy

### Statistical analysis

Data analysis was performed by means of SPSS version 13.0 software program (SPSS Inc., Chicago, IL, USA) through calculations of descriptive statistical methods (frequency, mean and standard deviation). McNemar’s test was used to compare preoperative and postoperative outcomes. P value <0.05 was considered statistically significant.

## Results

The septum was completely removed during the first hysteroscopy in 242 (92%) patients. A residual septum was seen in 21 (8%) patients with required additional intervention. The results of HSG after the second surgery in these 21 patients confirmed that the uterine septum was present in three patients, which were successfully removed after the third surgery. Three cases were complicated by small perforations, but did not need any additional treatment. One operation resulted in bleeding which was controlled by a Foley catheter. There were no cases of postoperative Asherman’s syndrome.

The mean age, body mass index (BMI), mean duration of infertility, other etiology of infertility and the number of patients with histories of assisted reproductive technology (ART) and IUI are shown in table 1. According to the results of HSG, 245 (93.2%) patients had partial septum, whereas 18 (6.8 %) patients had complete septum (Table 1).

**Table 1:** Patients’ clinical characteristics


	Primary	Secondary	Recurrent miscarriage	Total

**Patients**	196	52	15	263
Type of septum				
**Partial**	179 (91.3%)	51 (98.1%)	15 (100%)	245 (93.2%)
**Complete**	17 (8.7%)	1 (1.9%)	0	18 (6.8 %)
**Age (Y) (Mean ± SD)**	30.1 ± 5.9	32.2 ± 6.5	30 ± 4.4	30.1 ± 5.9
**BMI (Kg/m2) (Mean ± SD)**	27.5 ± 4.5	27.5 ± 3	28.5 ± 4.7	27.5 ± 3
**Infertility duration (Y) (Mean ± SD)**	7.6 ± 5.2	9.3 ± 6.5	-	7.95 ± 5.5
**Other etiologies of infertility**	152 (77.6%)	38 (73.1%)	-	190 (76.6%)
1 etiology	28 (18.4%)	11 (28.9%)	-	39 (20.5%)
> 1 etiology	124 (81.6%)	27 (71.1%)	-	151 (79.5%)
**Patients with history of ART**	33 (16.8%)	13 (25%)	0	46 (17.5%)
**Patients with history of COH+ IUI**	15 (7.7%)	4 (7.7%)	1 (6.7%)	20 (7.6%)


Postoperatively, outcomes of 203 out of 263 patients were analyzed, while 60 patients were lost in follow up. Out of 203 patients, there were 80 (39.4%) patients treated with ART, 27 (13.3%) patients treated with IUI, and 96 patients trying to get pregnant, naturally. Pregnancy was achieved in 80 (39.4%) out of 203 patients in the following conditions: 45 (56.2%) naturally, 25 (31.3%) after ART and 10 (12.5%) after IUI. From these patients, 10 (12.5%) pregnancies ended in miscarriages among whom 6 following ART, 1 after IUI and 3 women conceiving spontaneously. Table 2 shows the outcomes of pregnancies as follows: 1 (1.25%) was ectopic, 1 (1.25%) ended in preterm labor, and 68 (85%) resulted in term deliveries.

There were 52 patients with histories of 62 pregnancies (6 patients with history of miscarriage and preterm labor, 2 patients had history of ectopic pregnancy and miscarriage, while one with history of miscarriage, preterm labor and term delivery) resulting to the following conditions: 41 (20.2%) ended in spontaneous miscarriages, 7 (3.4%) were ectopic, 9 (4.4 %) resulted in preterm labor and 5 (2.5%) ended in term delivery. The postoperative outcome in this group resulted in 80 women who became pregnant with the following outcomes: 10 (4.9%) ended in spontaneous miscarriages, one (0.5%) ectopic pregnancy, one preterm labor (0.5%) and 68 (33.5%) with full term deliveries. Postoperatively, rates of miscarriage, preterm labor and ectopic pregnancy reduced as compared to before (p<0.0001, p=0.02, and p=0.07). Term deliveries increased significantly from 2.5% to 33.5% (p<0.0001,Table 3).

**Table 2:** Reproductive outcome of women after hysteroscopic septum resection


	Primary	Secondary	Recurrent miscarriage	Total

**Patients**	196	52	15	263
**Tend to get pregnant**	149 (76%)	41 (78.8%)	13 (86.7%)	203 (77.2%)
**Lost to follow up **	47 (24%)	11 (21.1%)	2 (13.4%)	60 (22.8%)
**Patients with ART**	61 (40.9%)	16 (39%)	3 (23.1%)	80 (39.4%)
**Patients with IUI**	19 (12.8%)	6 (14.6%)	2 (15.4%)	27 (13.3%)
**Pregnant**	57 (38.3%)	15 (36.6%)	8 (61.5%)	80 (39.4%)
Natural	27 (47.4%)	13 (86.7%)	5 (62.5%)	45 (56.2%)
After ART	21 (36.8%)	2 (13.3%)	2 (25%)	25 (31.3%)
After IUI	9 (15.8%)	0	1 (12.5%)	10 (12.5%)
**Miscarriages**	7 (12.3%)	3 (20%)	0	10 (12.5%)
**Ectopic pregnancies **	0	1 (6.7%)	0	1 (1.25%)
**Preterm labor**	0	1 (6.7%)	0	1 (1.25%)
**Term deliveries**	50 (87.7%)	10 (66.7%)	8(100%)	68 (85%)


**Table 3:** Comparison of pre- and post-operative outcomes


	miscarriage rate % (n)	Ectopic pregnancy rate % (n)	Preterm labor rate % (n)	Term delivery rate % (n)

**Before septum resection**	20.2% (41/203)	3.4% (7/ 203)	4.4% (9/203)	2.5% (5/203)
**After septum resection**	4.9% (10/203)	0.5% (1/203)	0.5% (1/203)	33.5% (68/203)
**P value**	<0.0001	0.07	0.02	<0.0001


## Discussion

In our study population, septum resection by hysteroscopy was followed by an obvious improvement in pregnancy outcome, while the preoperative rates of miscarriage, preterm labor and term delivery in the current study were 20.2, 4.4, and 2.5%, respectively. After hysteroscopic septum resection, the miscarriage and preterm labor rates dropped to 4.9 and 0.5%, respectively, and the term delivery rate increased to 33.5%. 

Our results are comparable with the findings of two other reviews of reproductive outcome before and after hysteroscopic septum resection (7, 18). Other researchers have reported postoperative miscarriage rates of 5-20% (14, 19) and term delivery rates of 62.8-87% (2-4, 20). This reproductive outcome improvement following septum resection might be related to an increased volume of uterine cavity, which creates an appropriate location for implantation and enhanced endometrial function via re-vascularization of uterine connective tissue (14, 21).

Hysteroscopic septum resection is a safe and effective procedure for achieving normal uterine shape (4, 22, 23). According to most studies, few complications have been observed in hysteroscopic septum resection (11, 22-24). This procedure is valuable in patients with recurrent miscarriage (23-25). However, there is a question regarding those patients with infertility (13, 26). It seems that a septate uterus is associated with sustaining a pregnancy, not infertility (2, 23); therefore, in patients with infertility, septum resection is performed to prevent miscarriage and preterm labor (22). Some studies have shown that uterine septate may be the cause of secondary infertility (2, 7, 10). In our study, 27 (47.4%) pregnancies in primary infertile patients and 13 (86.7%) pregnancies in secondary infertile patients were natural conception after septum resection, which raises the possibility of a septate uterus as a probable cause for infertility. Similarly, others have noted postoperative pregnancies in about 54% of infertile patients with uterine septum (25, 27). The mechanism of infertility and early pregnancy loss in women with septate uterus has not been ascertained (28, 29). Some investigators attribute this to the insufficient vascularization of the septum fibroelastic tissue and important ultrastructural changes of the septal endometrium during the preovulatory phase in comparison with the lateral wall of the endometrium, which creates a negative effect on implantation (2, 10, 29).

Based on our results, the rate of ectopic pregnancy decreased insignificantly after metroplasty from 3.4 to 0.5%. In one study (13), there was no significant change in ectopic pregnancy before and after septoplasty (2.6% vs. 2.3%). Although the risk for ectopic pregnancy increases in women with utero exposure to diethylstilbestrol (30), there is no study showing the relationship between septate uterus and tubal pregnancy.

Our postoperative miscarriage rate was 4.9% (10/203). Miscarriage was seen in a total of 6 out of 25 (24%) patients conceiving after ART, 3 out of 45 (6.7%) patients conceiving naturally, while 1 out of 10 patients after IUI (12.5%). This has presumably indicated that the reproductive outcome might have been influenced by the method of becoming pregnant as shown by Grimbizis et al. (2). 

During the first hysteroscopy, our results indicated lower prevalence of residual septum occurrence (8%) than the results reported by Kormanyos et al. (28) and Porcu et al. (31). However our rates were similar to published works of two other studies (32, 33).

In evaluating the recurrent miscarriage group, 61% tended to get pregnant. All patients conceiving after metroplasty ended in term deliveries. In a study by Venturali et al. (23), about 60% of the recurrent miscarriage group ended in term deliveries; although, the number of patients in this group (n=15) is not large enough to draw a conclusion.

## Conclusion

Hysteroscopic septum resection in the patients with infertility and recurrent miscarriage is a safe and effective method, followed by a significant improvement in the reproductive outcome by reducing miscarriage rate and preterm labor and increasing term delivery.
